# Supplementary material for: Plasticity without dislocations in a polycrystalline intermetallic
Source: Nat Commun. 2019 Aug 9;10:3587. doi: 10.1038/s41467-019-11505-1 (PMC6689057; doi:10.1038/s41467-019-11505-1)
Supplement: Supplementary file 1 — Supplementary Information [file 41467_2019_11505_MOESM1_ESM.pdf]

Supplementary Information

Plasticity without Dislocations in a Polycrystalline Intermetallic

Luo et al.

Supplementary Note 1| Empirical potential for $SmCo_5$

A highly optimized embedded-atom-method (EAM)[1,2] interatomic potential has been developed for the magnetic Co-Sm system. The potential is accurate in describing the energetics and phase behavior of complex Co-Sm intermetallic compounds as well as different allotropes of Co and Sm.

To develop the potential, we employed the force-matching method by fitting the potential to a large density functional theory (DFT) database established for Co-Sm. The methodology of the fitting procedure has been documented in previous publications[3,4]. In this work, we first carried out extensive DFT calculations to sample the potential energy surface of Co-Sm. The atomic configurations we selected in the potential development of Co-Sm include (i) equations of state (EOS) of known crystal structures of Co, Sm, and Co-Sm intermetallics; (ii) trajectories of selected crystals (intermetallics) during the heating and cooling cycles employing *ab initio* molecular dynamics (MD) (see, e.g., Supplementary Figure 1, for explanation); (iii) a comprehensive list of defect types of both pure Co and Sm, including point defects, surfaces, stacking faults, transition paths etc.; (iv) equations of state of amorphous Co_xSm_{1-x} configurations quenched from high temperature liquids. These atomic configurations were subjected to high-precision DFT total energy calculations, and in this way, we constructed a DFT database for Co-Sm, consisting of cohesive energies and stress tensors of all atomic configurations and forces on the atoms. The collected DFT data were slightly shifted and scaled to match the experimental data (e.g., lattice parameters and tabulated cohesive energies of Co and Sm)[4]. The potential was then optimized with the revised potfit code[2] by fitting the cohesive energies, forces and stress tensors of ~1500 atomic configurations (a total of 18,000 atoms) with proper fitting weights. Experimental elastic constants and gamma-point phonon frequencies of fcc cobalt were used to loosely constrain the fitting results. We used quintic splines to represent the density, pair, embedding functions in the EAM formalism, respectively. Lastly, the potential was refined through an iterative process by adding more DFT data into the fitting database to achieve self-consistency.

The DFT calculations were conducted with the plane-wave based Vienna *Ab-initio* Simulation Package (VASP)[5]. We used the projector augmented-wave (PAW) method[6] to describe the electron-ion interactions and the generalized gradient approximation (GGA)

for exchange-correlation functionals[7]. To account for the magnetism of the system, collinear spin-polarization calculations were performed on all configurations. The valance electrons of Co and Sm were specified as $3d^7 4s^2$ and $4f^6 5d^0 6s^2$, respectively. For high-precision DFT total-energy calculations, we typically used 3×3×3 Monkhorst-Pack[8] *k*-point grids and each atomic configuration typically contains 100-300 atoms.

To demonstrate the overall performance of the newly developed potential, we list the fundamental properties of Co-Sm as predicted by the EAM potential, and their comparisons with DFT calculations and experimental data, as shown in Supplementary Tables 1-3. Also shown are the cohesive energies of various types of Co, Sm and Co-Sm crystal structures as a function of volume, as illustrated in Supplementary Figures 2-4. It can be seen that the as-developed potential can satisfactorily describe the ground state properties of Co and Sm. The energetics of Co-Sm intermetallic compounds are correctly predicted by the EAM potential, and the energy differences between EAM and DFT calculations (Supplementary Table 3) are generally within several tens of meV, and in many cases, the energy differences are within several meV, indicating the high fidelity of the as-developed Co-Sm EAM potential. The goodness of the potential fitting of the Co-Sm system is found to be on par with pure fcc metals.

Supplementary Table 1| Crystal properties of Co. Predicted values of Co using the EAM potential compared with experimental properties, where E_c is the cohesive energy, C_{11}, C_{12} and C_{13} elastic moduli. ν_L and ν_T are Gamma-point longitudinal and transverse phonon frequencies, respectively.

Co	EAM	Experiment / DFT
Cohesive energy E_c (eV/atom) hcp, 300K a=2.507 Å; c=4.069 Å	-4.39	-4.39[a]
c/a ratio	1.612	1.623[b]
C_{11} (GPa) (fcc a=3.53 Å)	260	259[c], 225[d]
C_{12} (GPa) (fcc a=3.53 Å)	165	159[c], 160[d],
C_{44} (GPa) (fcc a=3.53 Å)	102	109[c], 92[d],
$\boldsymbol{\nu_L(X)}$ (THz) (fcc a=3.53 Å)	9.1	8.1[e]
$\boldsymbol{\nu_T(X)}$ (THz) (fcc a=3.53 Å)	6.22	5.8[e]
$\Delta E_{\mathrm{fcc-hcp}}$ (eV/atom)	0.014	0.016[f],0.012[g]
$\Delta E_{\mathrm{9R-hcp}}$ (eV/atom)	0.018	0.020[g]
$\Delta E_{\varepsilon-\mathrm{hcp}}$ (eV/atom)	0.063	0.051[g]

[a]Ref. 9
[b]Ref. 10
[c]Ref. 11
[d]Ref. 12
[e]Ref. 13
[f]Ref. 14
[g]DFT calculation from the present work

Supplementary Table 2| Crystal properties of Sm. Predicted values of the EAM potential for Sm compared with experimental properties, where E_c is the cohesive energy, B bulk modulus, E_v^f the formation energy of vacancy in α-Sm.

Sm	EAM	Experiment /DFT
Cohesive energy E_c (eV/atom) a=3.61 Å; c=26.220 (α-Sm, 9R)	-2.14	-2.14[a]
Bulk Modulus (GPa)	41	38[b]
$\Delta E_{9R-dhcp}$ (eV/atom)	-0.0002	-0.0005[c]
ΔE_{9R-hcp} (eV/atom)	0.002	0.004[c]
ΔE_{9R-tI2} (eV/atom)	0.008	0.012[c]
ΔE_{9R-bcc} (eV/atom)	0.005	0.012[c]
$\boldsymbol{E_v^f}$(eV)	1.04	1.16[c]

[a]Ref. 9
[b]Ref. 15
[c]DFT calculation in the present work

Supplementary Table 3| Crystal properties of Co-Sm compounds. Cohesive energies of Co-Sm intermetallic compounds evaluated with the EAM potential in comparison with DFT calculations at the ground state. ΔE refers to the difference between EAM and DFT calculations. The structures for the Co_xSm_{1-x} intermetallic compounds were taken from Ref. 16.

Phase	Pearson symbol	SGR symbol	Volume (Å^3/atom)	DFT (eV/atom)	EAM (eV/atom)	ΔE (eV/atom)
**Co**	hP2	$P6_3\bar{m}mc$	11.14	-3.40	-3.390	0.010
**$Co_{17}Sm_2$**	hP38	$P6_3\bar{m}mc$	12.78	-4.138	-4.138	0
**$Co_{17}Sm_2$**	hR57	$R\bar{3}m$	13.39	-4.150	-4.144	0.006
**Co_5Sm**	hP6	$P6/\bar{m}mm$	14.36	-4.130	-4.140	-0.010
**$Co_{19}Sm_5$**	hR72	$R\bar{3}m$	14.71	-4.024	-4.049	-0.025
**$Co_{19}Sm_5$**	hP48	$P6_3\bar{m}mc$	15.60	-4.046	-4.051	-0.005
**Co_7Sm_2**	hR54	$R\bar{3}m$	14.97	-4.021	-4.021	0
**Co_7Sm_2**	hP36	$P6_3\bar{m}mc$	14.79	-4.034	-4.034	0
**Co_3Sm**	hR36	$R\bar{3}m$	14.59	-3.983	-3.973	0.010
**Co_2Sm**	hR18	$Pm\bar{3}m$	15.87	-3.810	-3.812	-0.002
**Co_2Sm**	cF24	$Fd\bar{3}m$	15.78	-3.812	-3.813	-0.001
**Co_2Sm_5**	mS28	$C12/c1$	25.57	-2.971	-2.912	0.060
**$CoSm_3$**	oP16	$Pnma$	26.31	-2.866	-2.840	0.026
**α-Sm**	hP9	$R\bar{3}m$	31.95	-2.140	-2.134	-0.006

Supplementary Table 4| The energy maxima (EMs, in mJ/m^2) on the PESs of different slip systems of $SmCo_5$ and some hcp metals. The multiple symbols in pyramidal 2***c***+***a*** and ***c***+***a*** slip represent the EMs sequentially during the slip as also shown in Supplementary Figure 2.

Slip system	$SmCo_5$		Mg	$Mg_{47}Y$	Ti	Zr	Zn
	DFT	EAM	DFT				
Basal (***a*** slip) $(0001)\langle 11\bar{2}0\rangle$	2147	1982	325[a]<br>276[d]	214[d]	550[b]	460[b]<br>451[c]	380[b]
Pyr. (2***c***+***a*** slip) $(2\bar{1}\bar{1}1)\langle\bar{2}116\rangle$	2162 (α)<br>3328 (β)<br>43200 (γ)	1744 (α)<br>2454 (β)<br>65280 (γ)					
Pyr. (***c***+***a*** slip) $(2\bar{1}\bar{1}1)\langle\bar{1}2\bar{1}3\rangle$	16784 (α)<br>3840 (β)	29680 (α)<br>4000 (β)					
Pyr. (***a*** slip) $(10\bar{1}1)\langle\bar{1}2\bar{1}0\rangle$	13472	16944					
Pris. (***a*** slip) $(1010)\langle\bar{1}2\bar{1}0\rangle$	17497	22396					
Pris. (***c*** slip) $(1010)\langle 0001\rangle$	6369	7685					
Pyr. (***a*** slip) $(10\bar{1}1)\langle 11\bar{2}0\rangle$			343[d]	312[d]			
Pyr. (***c***+***a*** slip) $(11\bar{2}2)\langle 11\bar{2}3\rangle$			310 (α)[d]<br>570 (β)[d]	280 (α)[d]<br>585 (β)[d]			

[a]Ref. 17
[b]Ref. 18
[c]Ref. 19
[d]Ref. 20

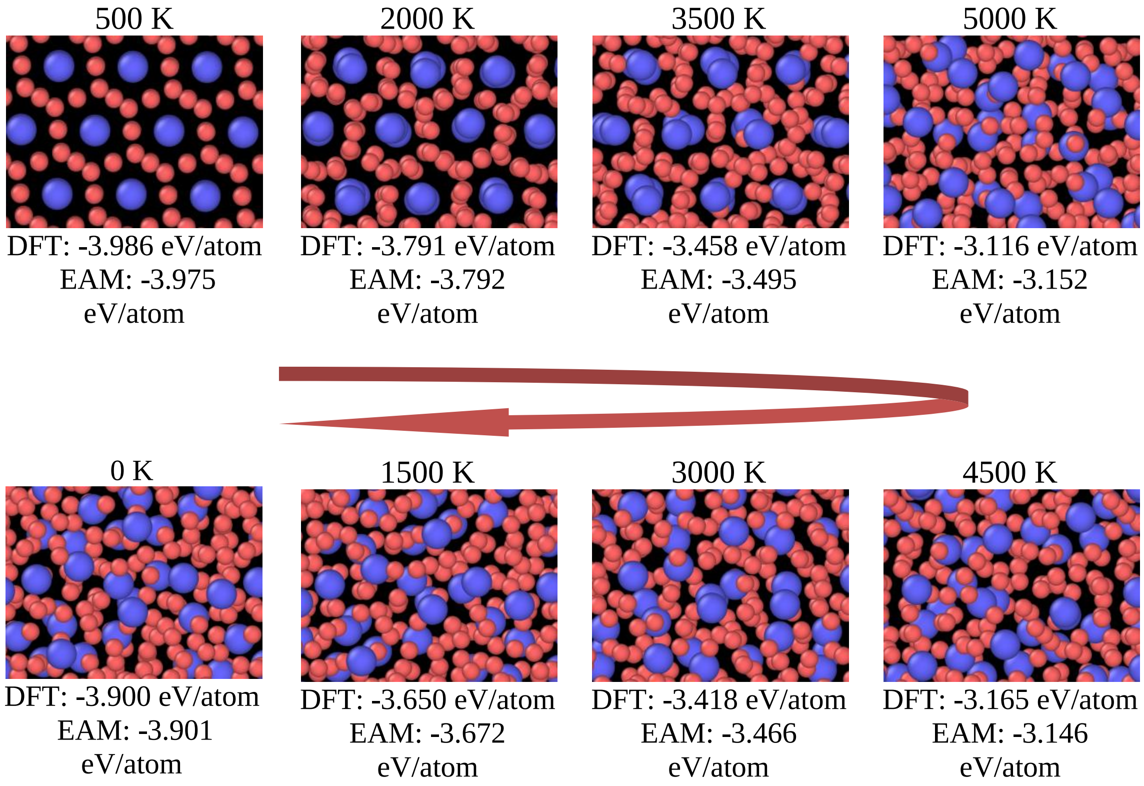

Supplementary Figure 1| Formation energies of different atomic configurations. Snapshots of Co_5Sm intermetallics taken from the heating and cooling cycle performed by *ab initio* MD (heating/cooling rate 5×10^{14} K/s). The atomic configurations were included in the DFT database for potential fitting. The comparisons of the formation energies evaluated by EAM and DFT treatments are shown for all configurations.

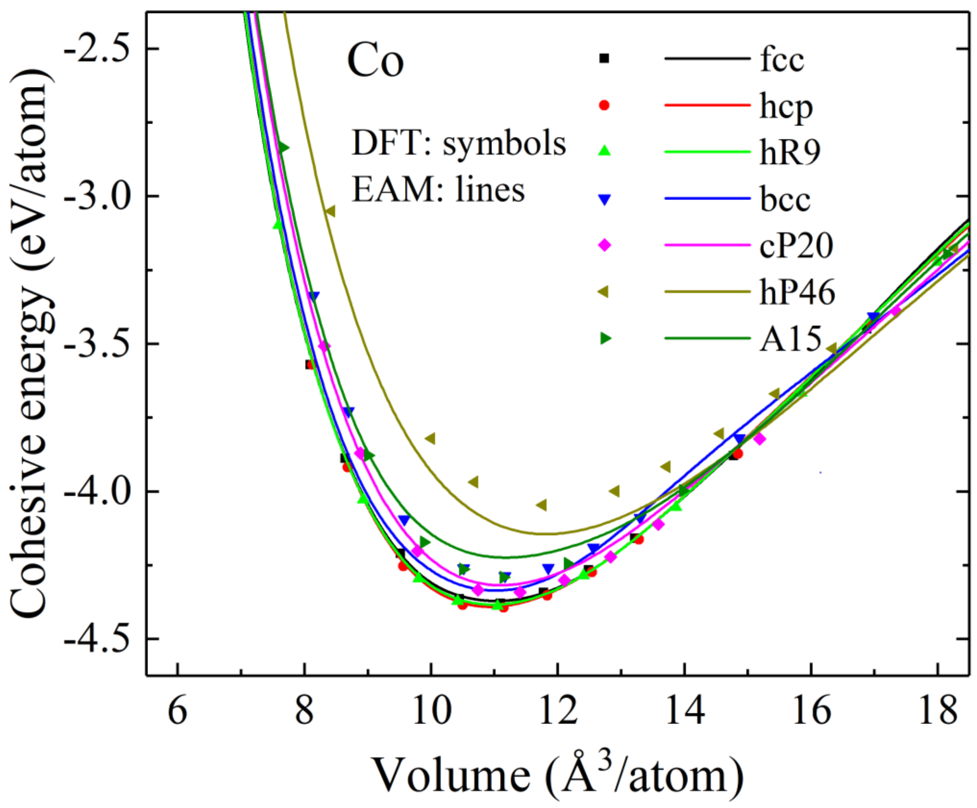

Supplementary Figure 2| Cohesive energies of Co. Comparison of DFT and EAM calculations of the cohesive energies of different crystal structures of Co as a function of volume.

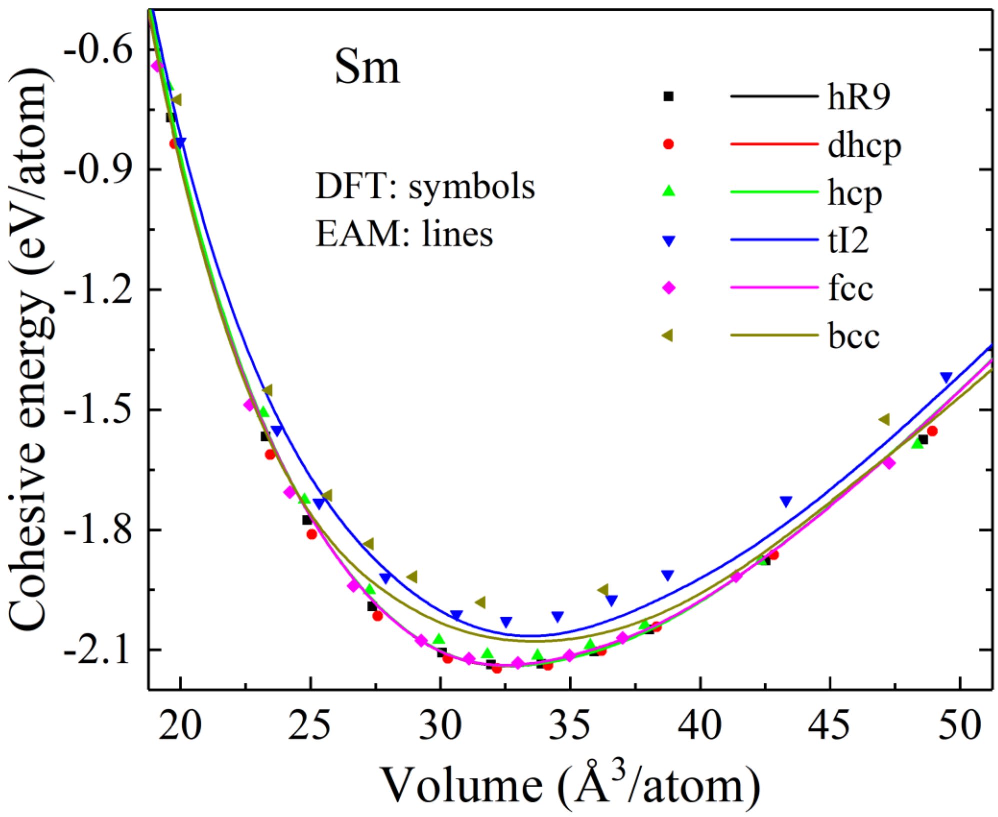

Supplementary Figure 3| Cohesive energies of Sm. Comparison of DFT and EAM calculations of the cohesive energies of different crystal structures of Sm as a function of volume.

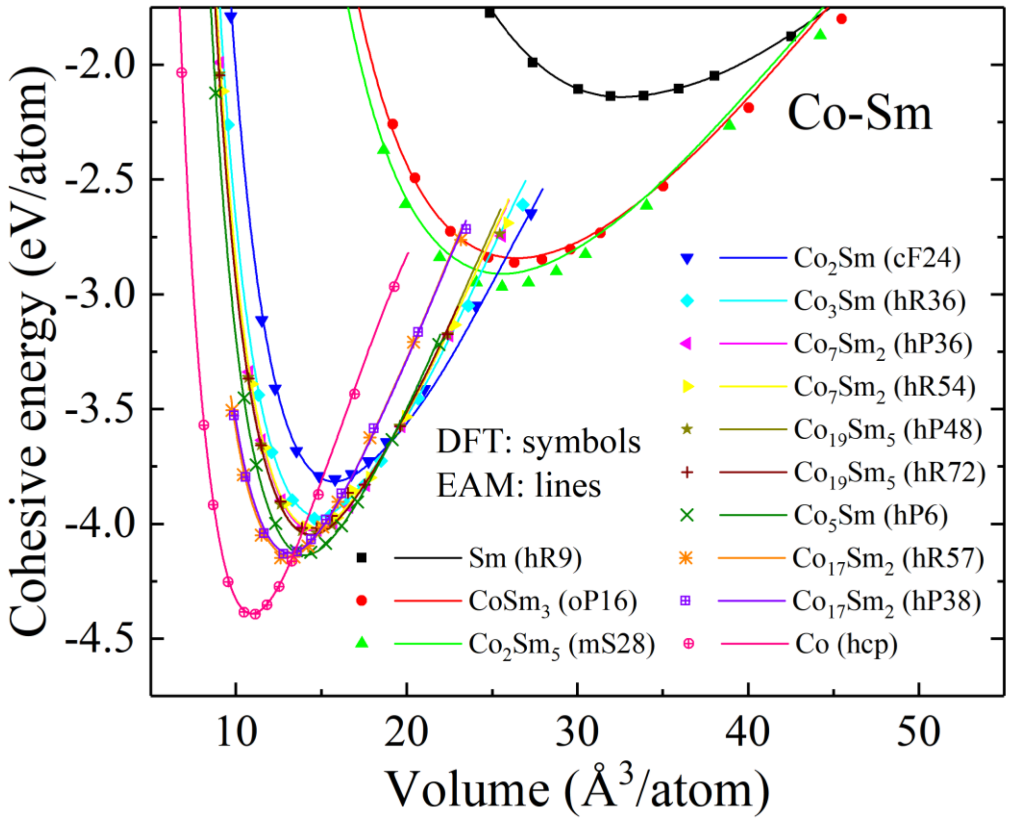

Supplementary Figure 4| Cohesive energies of Co-Sm compounds. Comparison of DFT and EAM calculations of the cohesive energies of different Co-Sm compounds as a function of volume.

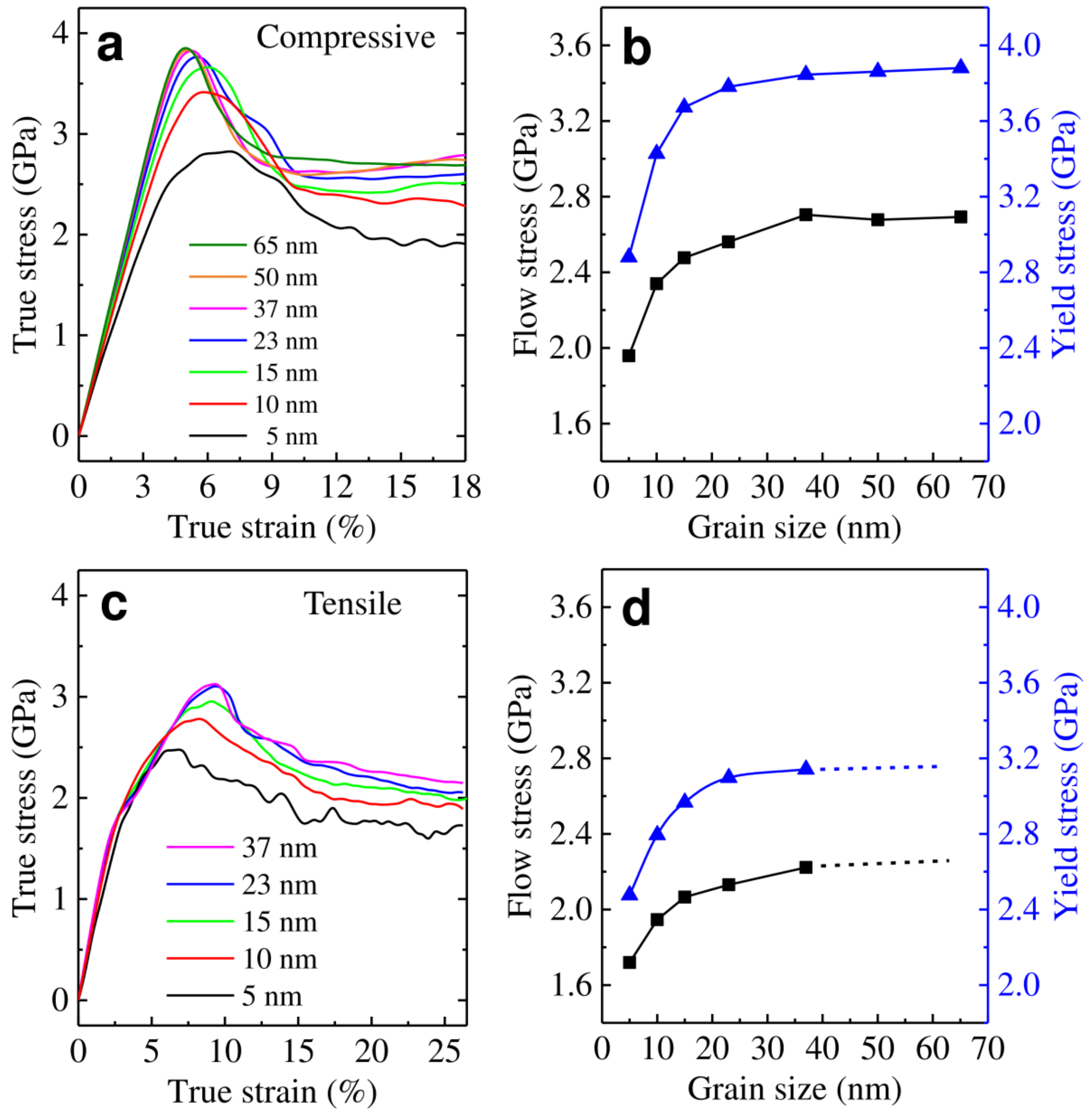

Supplementary Figure 5| Stress-strain curves and the corresponding yield and flow stresses with different grain sizes. (**a**) Stress-strain curves for compressive load with different grain sizes at strain rate of 10^8 s^{-1}. (**b**) The yield and flow stresses for compressive load as functions of grain size. (**c**) Stress-strain curves for tensile load of samples with different grain sizes at strain rate of 10^8 s^{-1}. (**d**) The yield and flow stresses for tensile load as functions of grain size. The flow stresses were calculated by considering the strain larger than 10.5% for compressive load and 18% for tensile load. Dashed lines represent trends for the flow stress and yield stress extrapolated to larger grain sizes. The softening after yield results from the release of accumulated local stress by initiation of plastic flow.

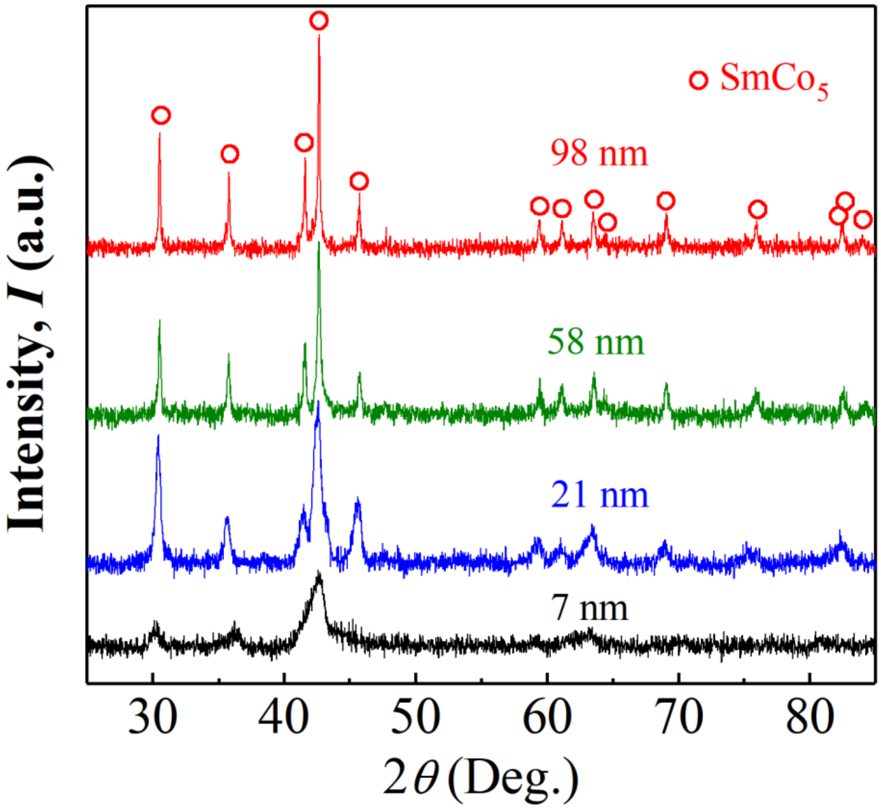

Supplementary Figure 6| X-Ray Diffraction (XRD) patterns of $SmCo_5$. XRD patterns of four $SmCo_5$ samples prepared with different techniques to control the grain size. No visible diffraction peaks of second phases are present.

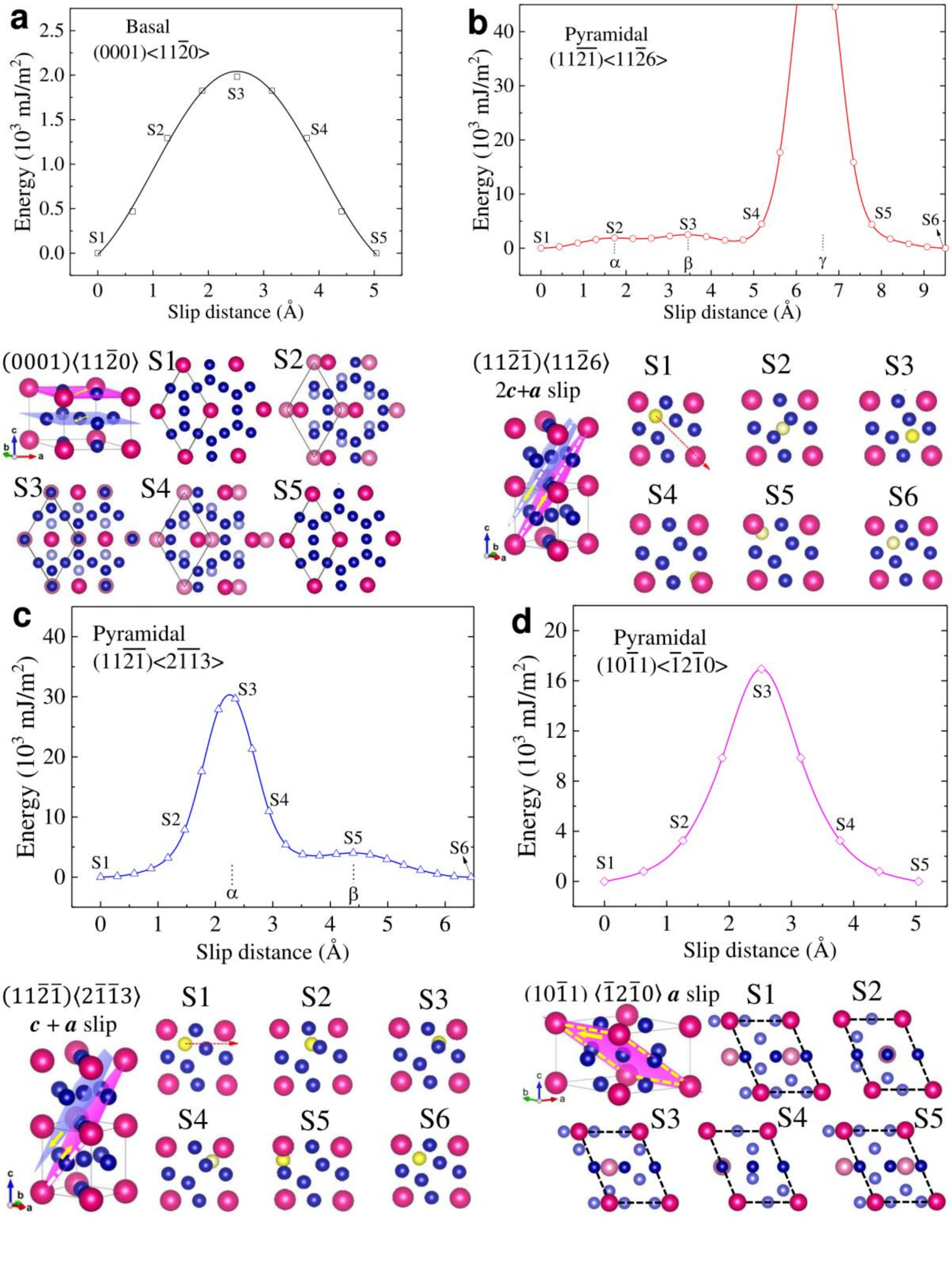

a
Basal
(0001)<11$\bar{2}$0>
Energy (10^3 mJ/m^2)
Slip distance (Å)
S1
S2
S3
S4
S5
b
Pyramidal
(11$\bar{2}\bar{1}$)<11$\bar{2}$6>
Energy (10^3 mJ/m^2)
Slip distance (Å)
S1
S2
S3
S4
S5
S6
α
β
γ
(0001)⟨11$\bar{2}$0⟩
(11$\bar{2}\bar{1}$)⟨11$\bar{2}$6⟩
2c+a slip
c
Pyramidal
(11$\bar{2}\bar{1}$)<2$\bar{1}\bar{1}$3>
Energy (10^3 mJ/m^2)
Slip distance (Å)
S1
S2
S3
S4
S5
S6
α
β
d
Pyramidal
(10$\bar{1}$1)<$\bar{1}$2$\bar{1}$0>
Energy (10^3 mJ/m^2)
Slip distance (Å)
S1
S2
S3
S4
S5
(11$\bar{2}\bar{1}$)⟨2$\bar{1}\bar{1}$3⟩
c + a slip
(10$\bar{1}$1) ⟨$\bar{1}$2$\bar{1}$0⟩ a slip

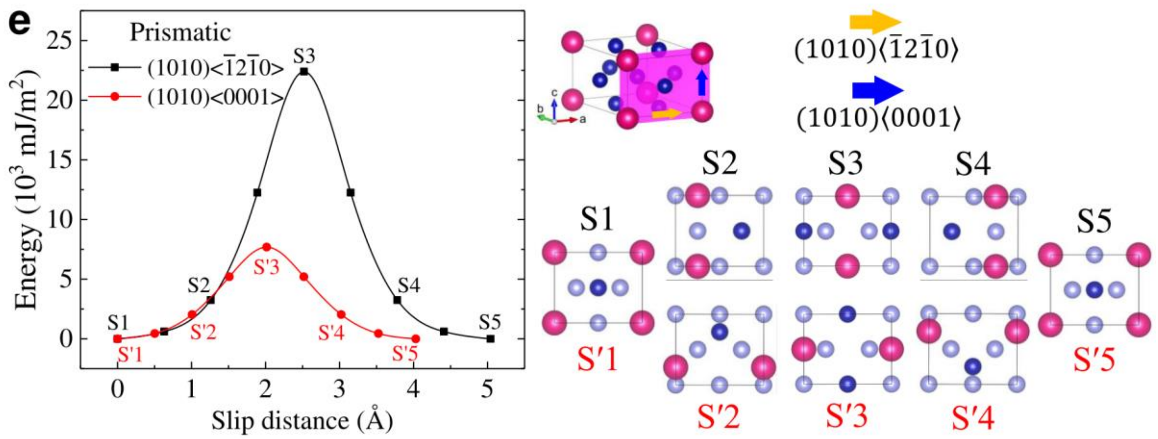

Supplementary Figure 7| The PESs of different slip systems calculated using EAM potential. The slip systems are chosen based on: (i) known slip systems for hexagonal closed packed metals, (ii) atomic displacements observed in our MD simulations, and (iii) by insight, i.e., by considering spacing of the planes and also the overlap between atoms during the slip so that the slip can possibly (partially) occur in deformation. (**a**) Basal slip $(0001)\langle 11\bar{2}0\rangle$. (**b**) Pyramidal 2$\boldsymbol{c}$+$\boldsymbol{a}$ slip $(11\bar{2}\bar{1})\langle 11\bar{2}6\rangle$. (**c**) Pyramidal $\boldsymbol{c}$+$\boldsymbol{a}$ slip $(11\bar{2}\bar{1})\langle 2\bar{1}\bar{1}3\rangle$. (**d**) Pyramidal $\boldsymbol{a}$ slip $(10\bar{1}1)\langle \bar{1}2\bar{1}0\rangle$. (**e**) Prismatic slips $(1010)\langle \bar{1}2\bar{1}0\rangle$ and $(1010)\langle 0001\rangle$. Detailed crystal structures during a slip are given along with the PESs and are labeled by symbols in accordance with those in the plot. The atoms beneath the top slip plane have lighter colors, except for the Co atoms colored in yellow for pyramidal $\boldsymbol{c}$+$\boldsymbol{a}$ and $\boldsymbol{a}$ slips, where there is only one atom beneath the top plane in the lattice period. Multiple energy maxima (EMs) are also pointed out by dashed lines and labeled by symbols as listed in Supplementary Table 1.

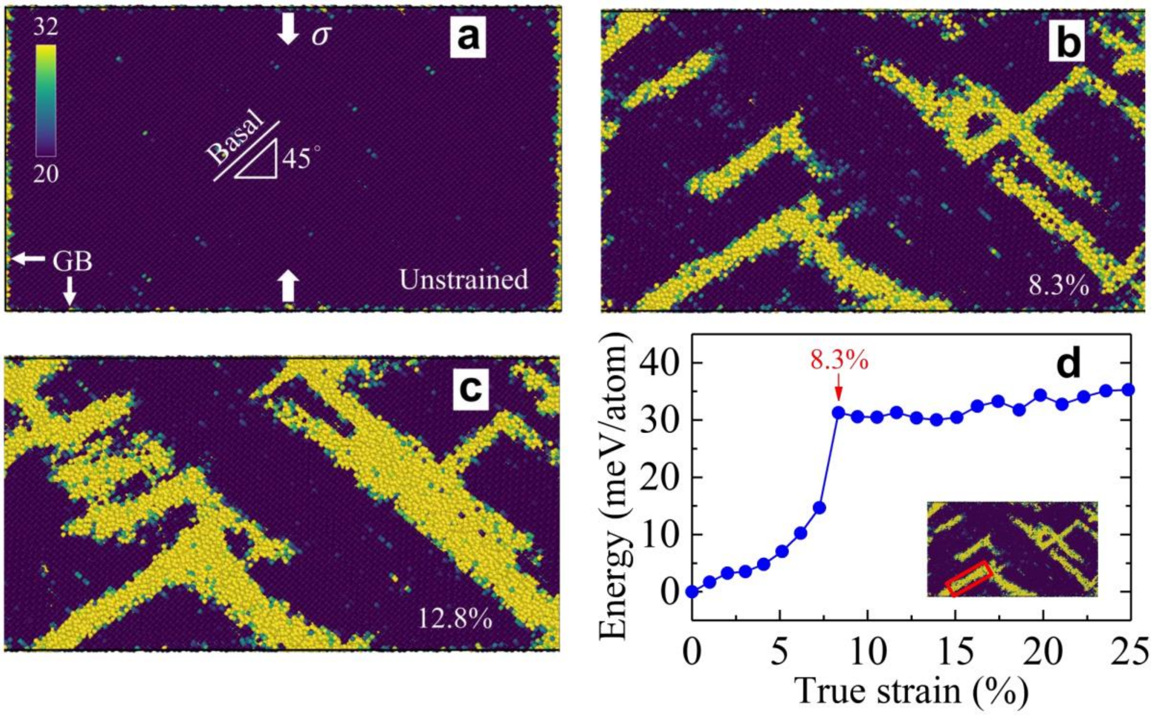

Supplementary Figure 8| Amorphous shear bands initiated inside an orthorhombic grain by strain. Atoms (only Sm atoms are considered) in the figures are colored by their centrosymmetry parameter. (**a**) The unstrained grain with the basal plane tilted to obtain a maximum resolved shear stress. (**b**) Amorphous shear bands (yellow atoms) initiated inside the grain at the strain of 8.3% lied along the planes with the largest resolved shear stress. (**c**) The shear bands expand, and the number of shear bands increases at the strain of 12.8%. (**d**) Energy of Sm atoms within a region where shear bands form as a function of strain. Sm atoms were selected in the red rectangle with their centrosymmetry parameter larger than 36 at the strain of 8.3% (inset).

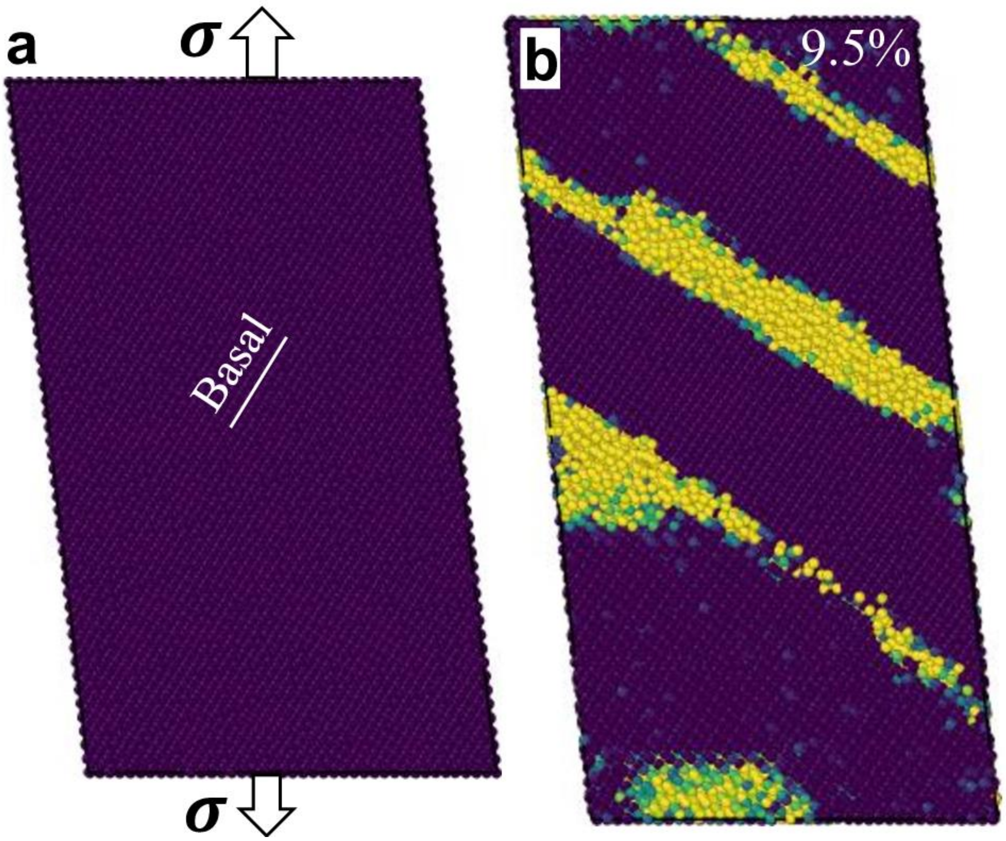

Supplementary Figure 9| Tensile deformation of a single crystal. (**a**)The supercell of the unstrained single crytal. (**b**) The single crystal undergoes a true tensile strain of 9.5%. The atoms (only Sm) are colored by centrosymmetry parameter. The amorphous shear bands are initiated in the prismatic planes since they are lying close to the plane with maximum shear stress.

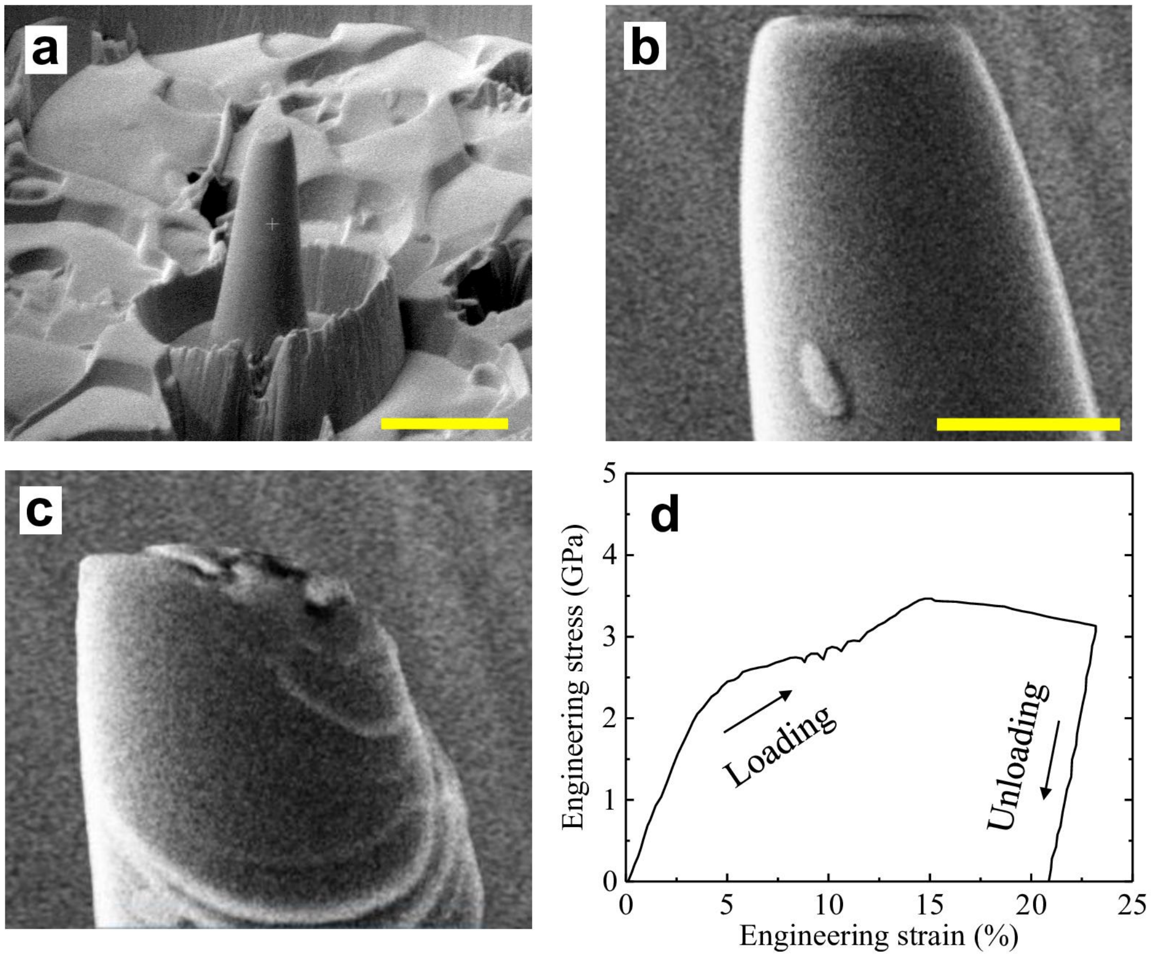

Supplementary Figure 10| Compression of micropillar $SmCo_5$. (**a**) The micropillar cut from a sample with grain sizes in the micrometer regime. Scale bar, 5 μm. (**b,c**) Compression of the micropillar using a diamond indenter leads to plastic shearing without failing by fracture. Scale bar, 1 μm. (**d**) The engineering stress-strain curve shows large plastic deformation without fracture up to 22% engineering strain.

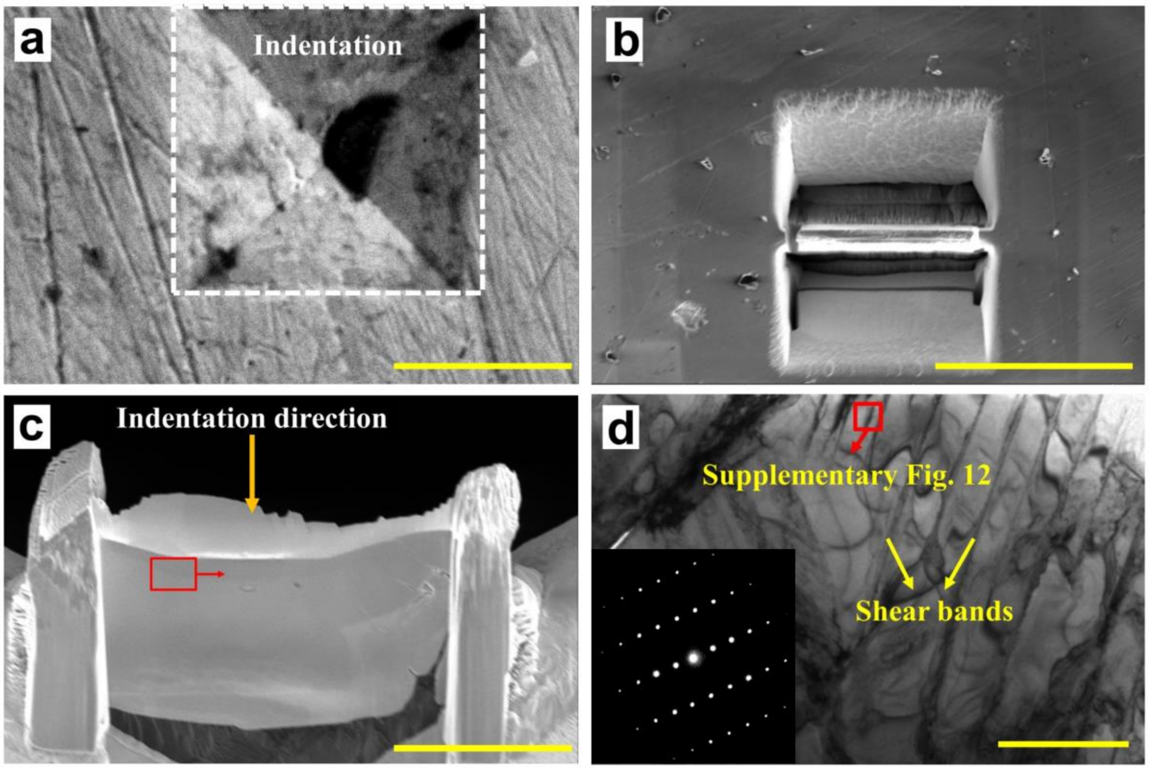

Supplementary Figure 11| TEM sample and shear bands. (**a-c**) The TEM sample cut from an indented area using focused ion beam. The final TEM sample has a thickness of about 100 nm. Scale bars, 10 μm (a), 30 μm (b) and 10 μm (c). (**d**) Magnified area from a rectangle in (**c**) shows multiple shear bands which are almost parallel to each other. The zone in the red square is selected for detailed characterization in Supplementary Fig. 12. Dark features between shear bands are due to variation in sample thickness, since they drift when the sample is tilted during TEM analysis. The inset shows the selected area diffraction pattern of the unsheared zone. Scale bar, 1 μm.

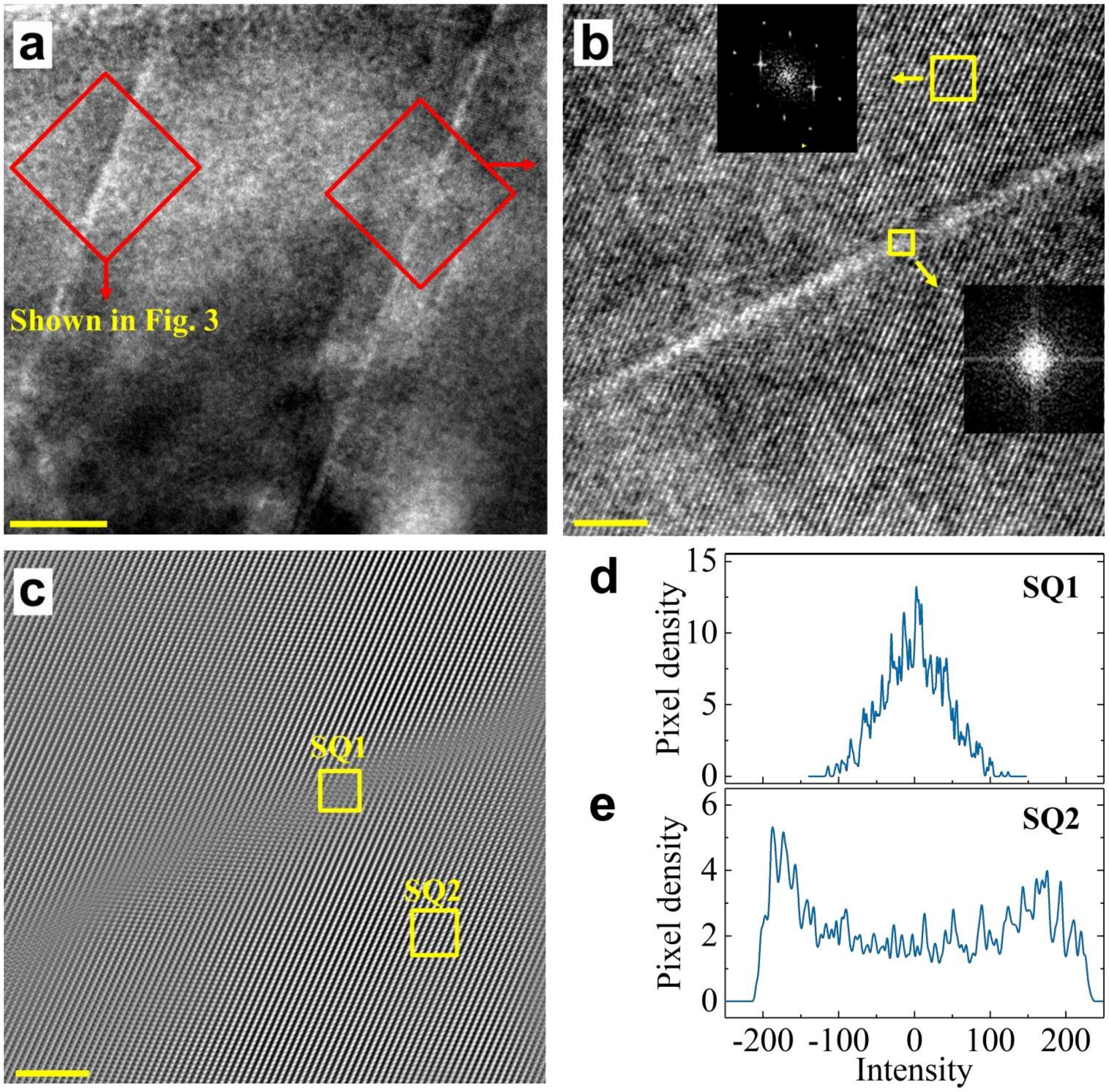

Supplementary Figure 12| Identification of amorphous shear bands in TEM sample. (**a**) Magnified high-resolution TEM image of the region marked in Supplementary Fig. 11d with a red rectangle. Scale bar, 20 nm. Two parallel shear bands are found. The area in the left red square was chosen for a detailed analysis in Fig. 3 of the main text. The area in the right rectangle is analyzed in (**b-e)**. (**b**) High-resolution TEM image of a shear band and its surrounding regions. The thickness of the shear band is about 2 nm. The lattice fringes shown in the shear band originate from overlap of amorphous shear band and lattice, as we found that they changed to be amorphous when the focus plane was adjusted. The insets show FFT patterns for the areas off the shear band and in the shear band, which further show that the off-band zone is crystalline while the in-band zone is amorphous. Scale bar,

5 nm. (**c**) The inverse FFT pattern of (**b**), in which the amorphous shear band results in a blurred stripe since only crystalline information is preserved in the inverse FFT. Scale bar, 5 nm. (**d,e**) Pixel distributions of intensity in the two squares from (**c**) are unimodal and bimodal for the shear band and its surrounding regions, respectively, confirming the amorphous and crystalline characters of these regions.

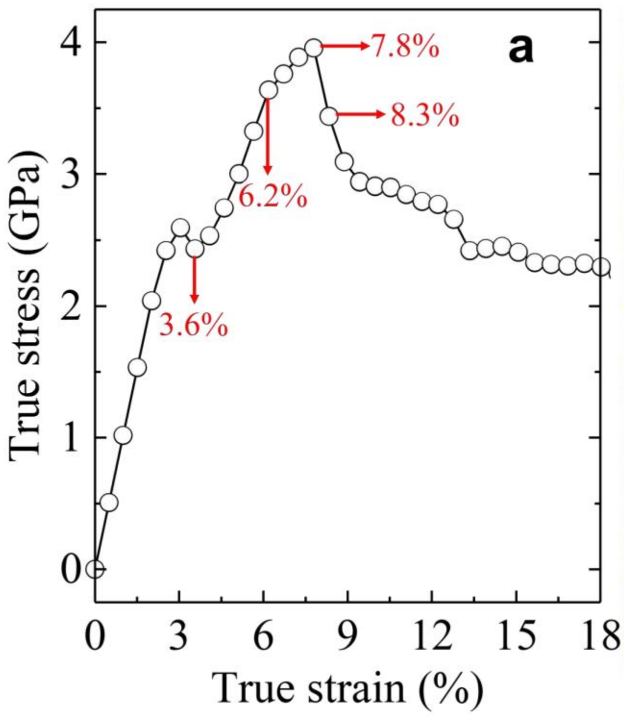

Supplementary Figure 13| Behavior of a pre-existing edge dislocation in an orthorhombic grain under compressive load. The pre-existing edge dislocation lies in the basal plane with a Burgers vector $\langle 11\bar{2}0\rangle/3$ and is first relaxed at 300 K. The basal plane has been titled to obtain a maximum resolved shear stress. The loading speed is the same as in polycrystalline samples. (**a**) The corresponding stress-strain curve with arrows indicating strains for which the atomic structures have been visualized in (**b-e**). (**b-e**) Evolution of the dislocation under a compressive load at strains ranging from 0 to 8.3%. Only Sm atoms are considered in the calculation of centrosymmetry parameter and are shown and colored here. The as-relaxed dislocation has a width of about 4 nm as shown in (**b**). The dislocation expands toward a grain boundary (which is in the right top corner) when the applied strain is lower than 6.2% (**c**). At the strain of 7.8%, the dislocation transforms to an amorphous shear band as can be seen from the amplified local atomic structure in the inset of panel (**d**). The amorphous shear band penetrates the grain after yielding at the strain of 8.3% (**e**). We have carried out analogous simulations using free boundary conditions in the two lateral directions (perpendicular to loading direction) and the mechanisms found in both types of simulations are qualitatively the same.

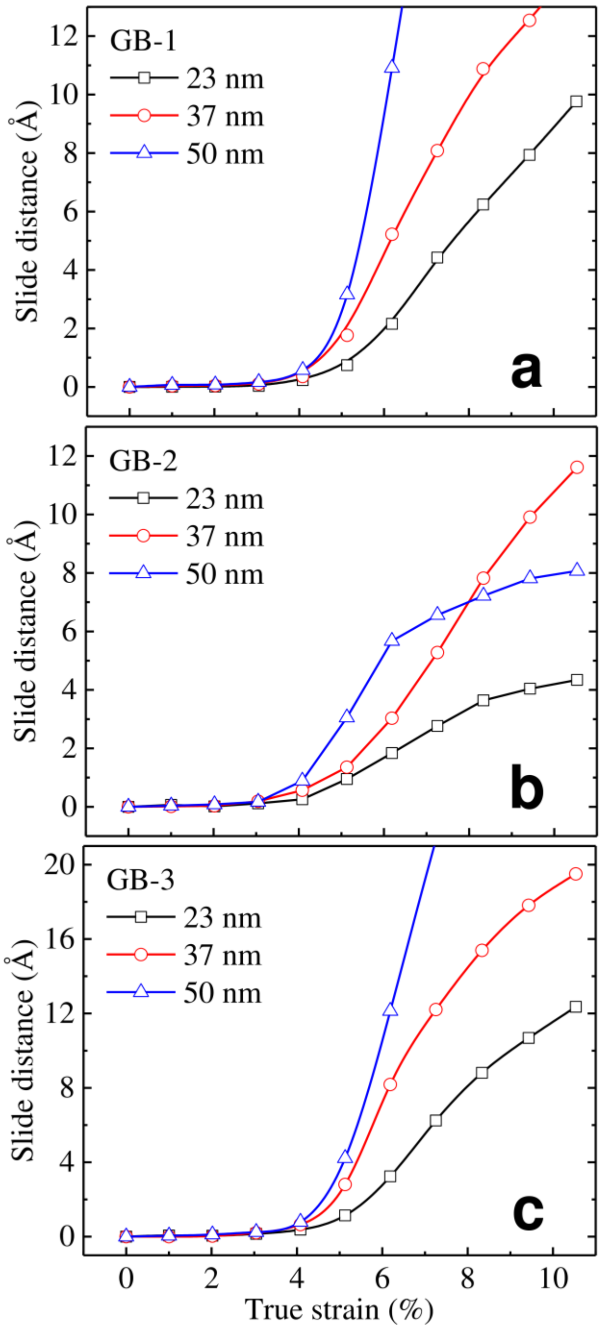

Supplementary Figure 14| Sliding distances as functions of the strain of three independent GBs that induce amorphous shear bands. (**a**) The GB sliding already shown in the main text (Fig. 4). (**b,c**) Other two examples of independent GBs that slide and lead to formation of amorphous shear bands. These GBs generally accommodate more strain for larger grain size as the slide distance is larger. For GB-2 in (**b**), the slide distance for the grain diameter of 50 nm increases slower after the strain of 6.2%. The reason is that there is competition from other activated shear bands in accommodation of strain.

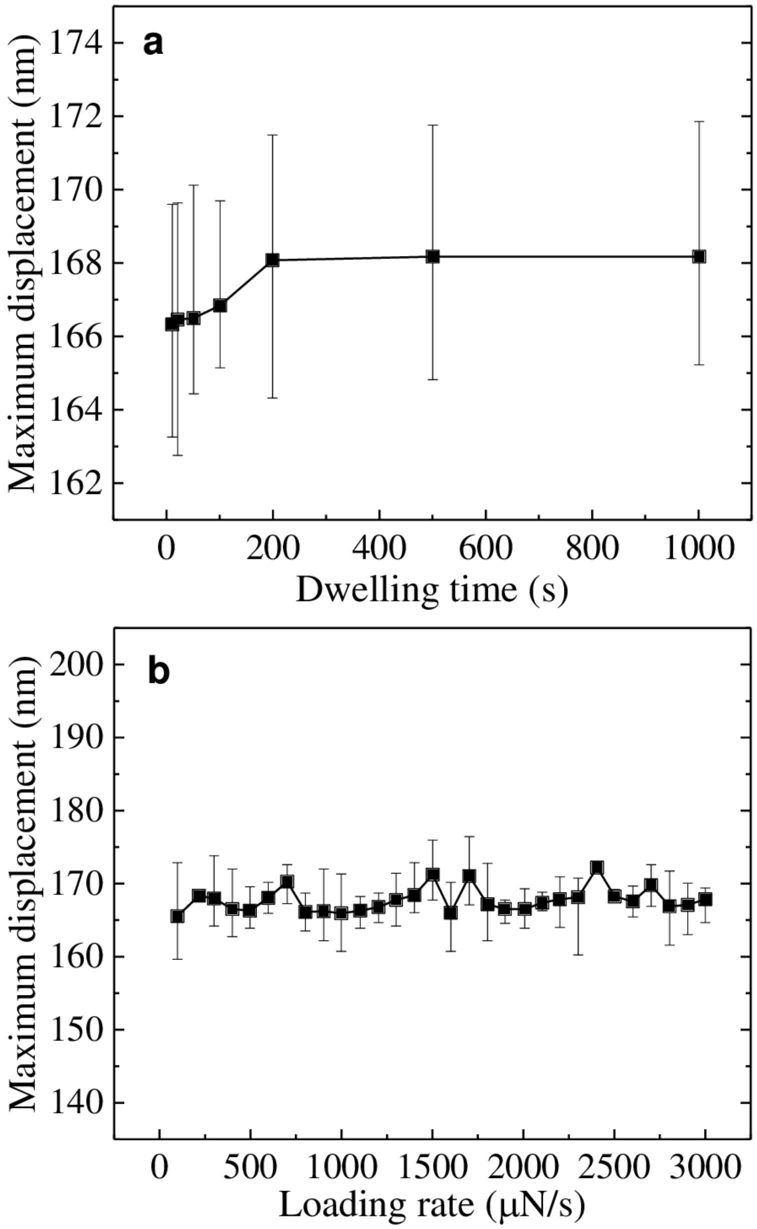

Supplementary Figure 15| Nanoindentation test of a strain-rate dependence (a) The maximum displacement of the indenter as a function of a dwelling time under a force of 6000 μN. (b) The maximum displacement of the indenter as a function of loading rate with a maximum force of 6000 μN. Error bars represent the standard deviation of the mean from the average values of the displacements. Experiments were carried out on a $SmCo_5$ sample with a 12 nm average grain size.

Supplementary references

1. Daw, M. S. & Baskes, M. I. Embedded-atom method: Derivation and application to impurities, surfaces, and other defects in metals. *Phys. Rev. B* **29**, 6443-6453 (1984).
2. Peter, B. & Franz, G. Potfit: effective potentials from ab initio data. *Modell. Simul. Mater. Sci. Eng.* **15**, 295 (2007).
3. Cheng, Y. Q., Ma, E. & Sheng, H. W. Atomic Level Structure in Multicomponent Bulk Metallic Glass. *Phys. Rev. Lett.* **102**, 245501 (2009).
4. Sheng, H. W., Kramer, M. J., Cadien, A., Fujita, T. & Chen, M. W. Highly optimized embedded-atom-method potentials for fourteen fcc metals. *Phys. Rev. B* **83**, 134118 (2011).
5. Kresse, G. & Joubert, D. From ultrasoft pseudopotentials to the projector augmented-wave method. *Phys. Rev. B* **59**, 1758-1775 (1999).
6. Blöchl, P. E. Projector augmented-wave method. *Phys. Rev. B* **50**, 17953-17979 (1994).
7. Perdew, J. P., Burke, K. & Ernzerhof, M. Generalized gradient approximation made simple. *Phys. Rev. Lett.* **77**, 3865-3868 (1996).
8. Monkhorst, H. J. & Pack, J. D. Special points for Brillouin-zone integrations. *Phys. Rev. B* **13**, 5188-5192 (1976).
9. Kittel, C. *Introduction to Solid State Physics, 8th Edition*. (Wiley, 2004).
10. Pearson, W. B. *A Handbook of Lattice Spacings and Structure of Metals and Alloys*. Vol. 2 (Pergamon Press Ltd., 1967).
11. Simons, G. & Wang, H. *Single Crystal Elastic Constants and Calculated Aggregate Properties*. (MIT Press, 1977).
12. Gump, J. *et al.* Elastic constants of face-centered-cubic cobalt. *J. Appl. Phys.* **86**, 6005-6009 (1999).
13. Shapiro, S. M. & Moss, S. C. Lattice dynamics of face-centered-cubic $Co_{0.92}Fe_{0.08}$. *Phys. Rev. B* **15**, 2726-2730 (1977).
14. Nishizawa, T. & Ishida, K. The Co (Cobalt) system. *Bulletin of Alloy Phase Diagrams* **4**, 387-390 (1983).
15. Brandes, E. A. & Brook, G. B. *Smithells Metals References Book*. 7th edn, (Oxford, 1992).
16. Villars, P. & Cenzual, K. (ASM International®, Materials Park, Ohio, USA, 2017/18).
17. Wang, C. *et al.* Generalized-stacking-fault energy and twin-boundary energy of hexagonal close-packed Au: A first-principles calculation. *Sci. Rep.* **5**, 10213 (2015).
18. Wu, D., Zhang, J., Huang, J. C., Bei, H. & Nieh, T. G. Grain-boundary strengthening in nanocrystalline chromium and the Hall–Petch coefficient of body-centered cubic metals. *Scr. Mater.* **68**, 118-121 (2013).
19. Hou, S., Lei, H. & Zeng, Z. Hydrogen influence on generalized stacking fault energies of Zr {0001} basal plane: a first-principles study. *RSC Advances* **6**, 54371-54376 (2016).
20. Pei, Z. *et al.* Ab initio and atomistic study of generalized stacking fault energies in Mg and Mg–Y alloys. *New J. Phys.* **15**, 043020 (2013).
